# Assessment of Left Ventricular Strain Echocardiography in Individuals with Hashimoto’s Thyroiditis and Its Association with Serum TIMP-1 Concentration

**DOI:** 10.3390/jcm14051705

**Published:** 2025-03-03

**Authors:** Irfan V. Duzen, Selcen Y. Tuluce, Sadettin Ozturk, Mert D. Savcılıoglu, Huseyin Goksuluk, Gokhan Altunbas, Mehmet Kaplan, Ertan Vuruskan, Suzan Tabur, Murat Sucu, Seyithan Taysi

**Affiliations:** 1 Department of Cardiology, Faculty of Medicine, Gaziantep University Sahinbey Education and Research Hospital, Gaziantep University, Gaziantep 27310, Turkey; gokhanaltunbas@yandex.com (G.A.); kardiomehmet27@hotmail.com (M.K.); ertanvuruskan@hotmail.com (E.V.); mmuratsucu@gmail.com (M.S.); 2 Department of Cardiology, Cardiology Clinic, Heart Izmir Clinic, Izmir 35610, Turkey; selcenyakar06@gmail.com; 3 Department of Endocrinology and Metabolic Disease, Gaziantep City Hospital, Gaziantep 27470, Turkey; sadettinozturk27@hotmail.com; 4 Department of Cardiology, Cardiology Clinic, Gaziantep City Hospital, Gaziantep 27470, Turkey; mdsavcilioglu@gmail.com; 5 Department of Cardiology, Cardiology Clinic, Bursa Anadolu Hospital, Bursa 16320, Turkey; dr.hgoksuluk@gmail.com; 6 Department of Endocrinology and Metabolic Disease, Faculty of Medicine, Gaziantep University Sahinbey Education and Research Hospital, Gaziantep University, Gaziantep 27310, Turkey; suzan2471@yahoo.com.tr; 7 Department of Medical Biochemistry, Faculty of Medicine, Gaziantep University Sahinbey Education and Research Hospital, Gaziantep University, Gaziantep 27310, Turkey; seytaysi@hotmail.com

**Keywords:** TIMP-1, Hashimoto’s thyroiditis, strain

## Abstract

**Background**: Hashimoto’s thyroiditis (HT), which is an autoimmune condition and the primary cause of hypothyroidism, has numerous impacts on the cardiovascular system. This research aimed to compare TIMP-1 levels and LV strain values in euthyroid HT, hypothyroid HT, and healthy control persons. **Materials and Methods**: This study included 40 hypothyroid HT patients, 42 HT patients who became euthyroid with thyroid hormone replacement therapy, and 40 healthy controls. All subjects had conventional echocardiography and STE. Global and segmental LV longitudinal strain values (LVGLS) were calculated. Participants’ blood was tested for TIMP-1, thyroid function, and anti-TPO. **Results:** Higher serum TIMP-1 levels were found in euthyroid and hypothyroid HT patients than in the control group. Additionally, patients with euthyroid and hypothyroid HT displayed lower segmental and global LV strain values than the control group. A negative correlation was observed between strain values and TIMP-1 and anti-TPO levels. No significant difference was observed in serum TIMP-1 and strain values between euthyroid and hypothyroid HT patients. Patients with hypothyroid HT exhibited impaired diastolic function and reduced ejection fraction when compared to both euthyroid HT and control groups. However, euthyroid HT patients and the controls had similar diastolic function and ejection fractions. **Conclusions**: Hashimoto’s thyroiditis causes impairment of LV strain, regardless of thyroid hormone levels. Additionally, the condition is associated with elevated TIMP-1 levels. The relationship between LV strain values and anti-TPO levels indicates that the autoimmune component of the disease may be responsible for the impaired LV strain.

## 1. Introduction

Hashimoto’s thyroiditis (HT), is a prevalent autoimmune disorder that is the primary cause of hypothyroidism. The pathologic features of lymphocytic infiltration and follicular destruction are the histological hallmark of autoimmune thyroiditis (AIT), that can lead to gradual atrophy and fibrosis in various cases. In about 20% of patients, AITDs are associated with other organ specific/systemic autoimmune disorders [1].

Thyroid hormones exert many effects on the cardiovascular system, both directly and at the gene expression level. Cardiovascular modifications that occur in hypothyroidism encompass reduced preload and cardiac contractility, increased afterload, elevated peripheral vascular resistance, a decrease in heart rate, and impairment of diastolic function [2]. While it is commonly thought that these effects can be mitigated through thyroid hormone replacement therapy, the extent to which this treatment can reverse all of the adverse effects of HT remains a subject of debate as the condition has an autoimmune component.

Myocardial deformation is a key measurement in several cardiac conditions. It is obtained by strain and speckle tracking echocardiographic imaging, providing valuable diagnostic and prognostic information. The left ventricular global longitudinal strain (LVGLS) obtained by these echocardiographic techniques allows the detection of subtle myocardial dysfunction in various diseases [3,4,5,6].

Cardiac involvement frequently occurs in autoimmune diseases, with myocardial fibrosis serving as a significant mechanism underlying cardiac dysfunction induced by autoimmune processes. The chronic inflammatory state typically associated with autoimmune diseases appears to promote the development of both myocardial inflammation and fibrosis [7]. Research has demonstrated that the assessment of global and segmental strain values through speckle tracking echocardiography correlates with late gadolinium enhancement (LGE) observed in cardiac magnetic resonance imaging (MRI). Furthermore, a decline in segmental strain has been shown to signify the presence of myocardial fibrosis and subsequent myocardial dysfunction. Speckle tracking echocardiography (STE) has also been found effective in predicting cardiovascular adverse events in the follow-up of autoimmune diseases [8].

Matrix metalloproteinases and their tissue inhibitors play an important role in LV remodeling. It has been shown that a tissue inhibitor of metalloproteinase-1 (TIMP-1) can lead to collagen deposition and fibrosis in the myocardium, and therefore a reduction in LV systolic function. These effect is observed even in the early stages of cardiac remodeling, before prominent LV failure develops [9,10].

In the present study, we compared the blood TIMP-1 levels and LV functions using strain echocardiography among hypothyroid patients with a new diagnosis of HT, HT patients who had been euthyroid for a minimum of six months, and healthy control subjects.

## 2. Study Population

Fifty hypothyroid patients with a new diagnosis of HT and 59 patients with HT who had been euthyroid for at least 6 months were screened. A diagnosis of HT required the presence of circulating concentration of the anti–thyroid peroxidase antibody (TPO-Ab), thyroid stimulating hormone TSH, and clinical and biochemical signs, such as the classical features on a thyroid ultrasound. Patients with known coronary artery disease (*n* = 4), hypertension (*n* = 6), diabetes mellitus (*n* = 5), heart failure (*n* = 1) or moderate/severe valvular heart disease (*n* = 2), pregnancy (*n* = 1), patients with any other known autoimmune disorder (*n* = 4), lung disease (*n* = 2), chronic kidney disease (*n* = 1), and patients with cancer (*n* = 1) were excluded from the study. The remaining 40 hypothyroid patients with a new diagnosis of HT and 42 euthyroid HT patients were included in this study. Forty healthy individuals with normal thyroid function tests (TFT) without any known disease formed the control group.

## 3. Echocardiographic Study

### 3.1. Conventional Echocardiography

The subjects were imaged in the left lateral decubitus position. All subjects underwent two-dimensional, pulsed/continuous, M-mode, color flow Doppler echocardiographic examinations using a commercially available machine (GE Vivid E9, Harten, Norway) with a 2–4 MHz phased-array transducer. Left ventricular end-diastolic diameter, left atrial antero-posterior diameter, interventricular septum, and posterior wall thickness were obtained from a parasternal long-axis view, as recommended by the American Society of Echocardiography [11,12]. Simpson’s rule was used to estimate the left ventricular ejection fraction (LVEF). For the assessment of the diastolic function of the left ventricle (LV), a sample volume (1–3 mm) of the pulsed-wave Doppler was placed on the mitral leaflet tips, and mitral diastolic flow profiles were obtained in the apical four-chamber view. The peak flow velocities in the early diastole (E-wave) and late diastole (A-wave), E/A ratio, and E velocity deceleration time were recorded. Tissue Doppler imaging was used to obtain LV myocardial velocities in the apical four-chamber view by placing a sample volume at the septal and lateral segments of the mitral annulus during the early and late diastole (e′ and a′). Then, the E/e’ ratio was calculated. The left atrial volume (LAV) was calculated in four- and two-chamber views using Simpson’s rule and then indexed to body surface area to obtain the left atrial volume index (LAVI).

### 3.2. Two-Dimensional Speckle Tracking Echocardiography

For the assessment of LV longitudinal strain (LS), a dedicated Automated Function Imaging (AFI) protocol was employed. Two-dimensional grayscale images were acquired in the standard 3 apical views (apical 2-chamber, apical 3-chamber, and apical 4-chamber) at a frame rate of 70–90 frames/sec, and three cardiac cycles were recorded. The EchoPAC PC (Version 8.0. GE Healthcare, Horten, Norway) software package was utilized to analyze the acquired images. Utilizing this method, the end-systolic frame was defined in the apical long-axis view. The closure point of the aortic valve was demarcated, and the time interval between the R wave and aortic valve closure was measured by the software. This interval served as a reference for the echocardiographic loops. The tracked area was identified semiautomatically after selecting two basal corners at the level of the mitral annulus and a third point in the apex, with optional manual correction. On each apical view, the LV walls were divided into six segments, and the strain value and quality of tracking were subsequently assessed for each LV segment. The mean LS value was calculated independently in each of the three views. The LV global longitudinal strain (LVGLS) value was calculated as the arithmetic mean of the 3 values. Longitudinal strain was expressed as a negative value; a more negative or a higher absolute value indicated a greater extent of LS, pointing out a better LV function. In populations of healthy individuals, standardized assessments of LVGLS can vary across different manufacturers. In our investigation, the average LVGLS recorded was −20.5, aligning with previously documented normal LVGLS values for GE [13]. One significant characteristic of strain echocardiography is its dependable repeatability of measurements. In this investigation, we assessed both intraobserver and interobserver variability for segmental strain values corresponding to the apex, anterior wall, inferior wall, lateral wall, and septum, as well as for the global longitudinal strain (GLS) values in A2C, A3C, A4C, and LVGLS. The evaluation was conducted using the intraclass correlation coefficient (ICC). The results indicated an ICC value of no less than 0.954, which implies that the intraobserver and interobserver variability is quite good.

### 3.3. Venous Blood Sampling for TIMP-1

All venous blood samples were collected after an overnight fast. Blood samples were collected in tubes containing heparin. Serum samples were removed by centrifugation for 10 min at 4000× *g* rpm and the samples were stored at –80 °C before performing assays. TIMPs levels were measured with an ELISA reader (Biotekk ELx800, Winooski, VT, USA) and the color intensity formed by the enzyme-linked immunosorbent assay (ELISA) method using (Rel-Assay, Gaziantep, Turkey). Results are expressed as ng/mL.

### 3.4. Statistical Analysis

Statistical analysis was performed using the MedCalc^®^ Statistical Software version 19.7.2 (MedCalc Software Ltd., Ostend, Belgium; https://www.medcalc.org; 10 July 2022). The normality of continuous variables was assessed with Shapiro–Wilk’s test. Descriptive statistics were reported as mean, standard deviation, median, and interquartile range. Categorical variables were expressed as frequencies (n) and percentages (%). Chi-square tests were used to compare categorical variables. The Kruskal–Wallis test was employed to compare continuous variables among more than two independent groups. A Mann–Whitney U test with Bonferroni correction was used for post hoc comparisons. Pearson’s correlation coefficient was used to investigate correlations between normally distributed continuous variables, and Spearman’s rank coefficient of correlation (Rho) was used for non-normally distributed data. A result was considered statistically significant when its two-sided *p* value was less than 0.05.

## 4. Results

Comparisons among the study groups in terms of demographic characteristics, TFT results, and TIMP-1 levels are shown in Table 1. There were no significant differences among the euthyroid HT, hypothyroid HT, and control groups with respect to mean age (*p* = 0.987) and female sex ratio (*p* = 0.774). As expected, compared to the euthyroid HT and control groups, free T3 and T4 levels were lower in the hypothyroid HT group (both *p* values < 0.001). A significant difference was also observed in TSH levels among the groups (*p* < 0.001), which was caused by a higher TSH level in the hypothyroid HT group compared to the euthyroid HT and control groups.

The levels of TIMP-1 varied significantly between the groups (*p* < 0.001). The difference resulted from higher TIMP-1 levels in the hypothyroid HT and euthyroid HT groups versus the control group (both *p* values < 0.001). However, there were no significant differences between the euthyroid and hypothyroid HT groups in terms of TIMP-1 level (*p* = 1.0).

Table 2 shows statistical comparisons among the control, hypothyroid HT, and euthyroid HT groups for standard echocardiographic, Doppler, and tissue Doppler echocardiography indices. Compared to the control group, significant increases in the left atrium (LA) diameter, LV end-diastolic diameter (LVEDD) (both *p* values *p* < 0.001), and LV end-systolic diameter (LVESD) (*p* = 0.005) and a significant decrease in LVEF values (*p* = 0.027) were detected in hypothyroid HT patients. The E/e′ ratio was higher in hypothyroid HT patients compared to the controls (*p* < 0.001). Euthyroid HT patients exhibited higher LA diameters (*p* = 0.011) and higher LVEDDs (*p* = 0.007), but similar LAVI (*p* = 0.123), LVESD (*p* = 0.986), LVEF (*p*= 0.168), and E/e′ ratio (*p* = 0.111) values compared to the controls.

A significant difference was noted between the euthyroid HT patients and hypothyroid HT patients, with euthyroid HT patients exhibiting lower LVEDD (*p* = 0.029), LVESD (*p* < 0.001), and posterior wall thickness (*p* = 0.001) and higher LAVI (*p* < 0.001) values. The E/e′ ratio was numerically higher in hypothyroid HT patients compared to euthyroid patients. However, this difference did not reach statistical significance (*p* = 0.069).

Table 3 presents the statistical comparisons of the segmental and global strain measurements among the groups. While the hypothyroid HT and euthyroid HT groups predominantly exhibited lower LV segmental strain values compared to the controls (with the exception of the basal posterior septum), no statistically significant difference was observed between the hypothyroid and euthyroid HT groups in segmental strain values, except for the basal anterior septum, mid inferior wall, and basal lateral wall of the LV. Global longitudinal LV strain values were lower in the hypothyroid HT and euthyroid HT groups compared to the controls (both *p* values < 0.001). However, no significant difference was detected between the hypothyroid and euthyroid HT groups (*p* = 0.958).

**Figure 1:**
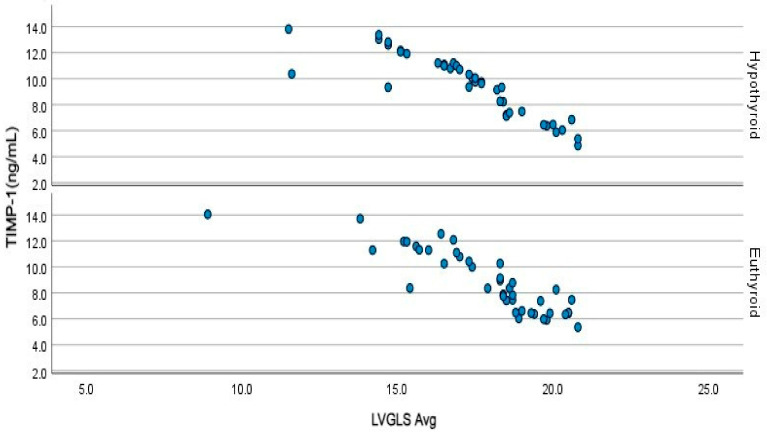
Negative correlations between TIMP-1 and the left ventricular global longitudinal strain values in hypothyroid and euthyroid Hashimoto patients. TIMP-1: Tissue inhibitor of metalloproteinase-1 LVGLS: left ventricular global longitudinal strain. Blue dots represent TIMP-1 levels.

**Figure 2:**
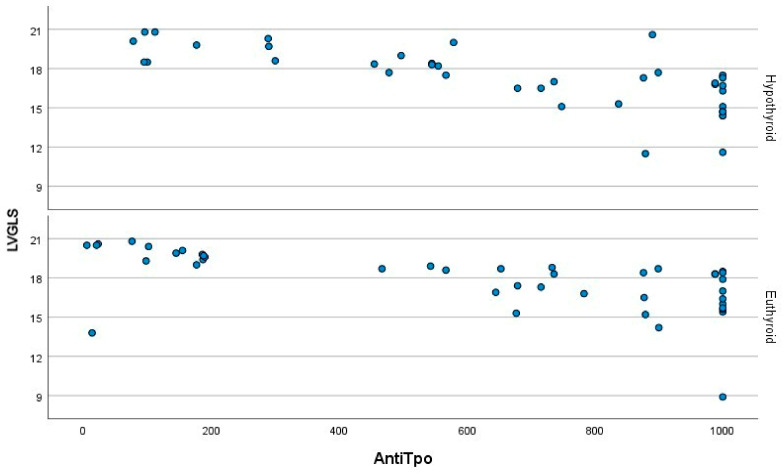
Negative correlations between anti-TPO levels and the left ventricular global longitudinal strain values in hypothyroid and euthyroid Hashimoto patients. LVGLS: left ventricular global longitudinal strain, Anti-TPO: Thyroid peroxidase (TPO) antibody. Blue dots represent anti-TPO levels.

**Figure 3:**
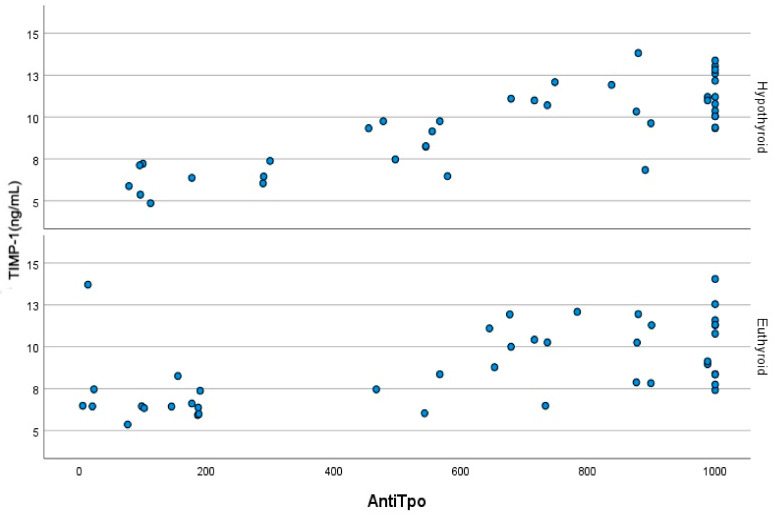
Positive correlations between anti-TPO levels and the TIMP-1 values in hypothyroid and euthyroid Hashimoto patients. TIMP-1: Tissue inhibitor of metalloproteinase-1, Anti-TPO: Thyroid peroxidase (TPO) antibody. Blue dots represent TIMP-1 levels.

Figure 1 illustrates the correlation between TIMP-1 levels and LVGLS. A strong negative correlation was also observed between TIMP-1 levels and LVGLS in both the euthyroid (r = −0.889, *p* < 0.001) and hypothyroid patients (r = −0.880, *p* < 0.001) with HT. As depicted in Figure 2, a strong negative association was evident between anti-TPO and LVGLS in both the euthyroid (r = −0.702, *p* < 0.001) and hypothyroid (*p* < 0.001) HT patients. Figure 3 demonstrates the presence of positive correlations between anti-TPO and TIMP-1 levels in both the euthyroid (r = 0.577, *p* < 0.001) and hypothyroid (r = 0.752, *p* < 0.001) patients with HT.

## 5. Discussion

In the current study, impaired LVGLS was observed in euthyroid and hypothyroid patients with HT compared to healthy controls, and impaired LVGLS exhibited a negative correlation with TIMP-1 and anti-TPO values. While hypothyroid HT patients showed slightly higher TIMP-1 levels in comparison to their euthyroid counterparts, the difference was not statistically significant. Although LVGLS was numerically lower in the hypothyroid HT group than in the euthyroid HT group, no significant difference was detected between the two groups. E/e′ ratio, an indirect marker of elevated LV filling pressure, was higher in the hypothyroid HT patients compared to the controls. Hypothyroid HT patients exhibited higher LV end-diastolic and end-systolic diameters in comparison to the euthyroid patients and healthy controls. Although LVEF was reduced in the hypothyroid HT patients compared to the control group, there was no significant difference in LVEF between the euthyroid HT group and the control group, or between the hypothyroid HT group and the euthyroid HT group.

Thyroid hormones regulate the expression of genes involved in the preservation of cardiac function, including downregulation of myosin heavy chain-β (*MYH7*) and phospholamban (*PLN*) and upregulation of myosin heavy chain-α (*MYH6)*, sarcoplasmic/endoplasmic reticulum calcium ATPase 2a (*SERCA2a*), and Na^+^/K^+^ ATPase. Consequently, hypothyroidism results in adverse transcriptional alterations that ultimately lead to impaired systolic and diastolic functions and reduced cardiac output. The upregulation of *SERCA2a* and downregulation of PLN in response to thyroid hormones enhance the reuptake of calcium into the sarcoplasmic reticulum, leading to improved ventricular relaxation in diastole. Specifically, patients with hypothyroidism may exhibit impaired ventricular relaxation due to reduced *SERCA2a* gene expression, increased PLN expression, and associated reductions in intracellular calcium reuptake as well as reductions in cardiac index and LV stroke volume both at rest and during exercise [14,15]. Consistently, in our study, diastolic dysfunction characterized by high IVRT and deceleration time, increased E/E’ ratio, increased left atrial volume index (LAVI), and low E-wave and high A-wave amplitudes were observed in hypothyroid patients with HT. In the same patients, a lower LVEF was detected in comparison with euthyroid HT patients and the control group. The euthyroid HT group showed improvements in diastolic parameters except for isovolumic relaxation time (IVRT) and had LVEF values similar to those of the control group.

Hashimoto’s thyroiditis is an autoimmune disease, and elevated anti-TPO is detected nearly in all affected patients [16]. In certain cases, the disease may be associated with nonendocrine disorders including alopecia areata, vitiligo, celiac disease, and autoimmune gastritis with vitamin B12 deficiency, which occur as a component of autoimmune polyglandular syndromes [17]. Despite thyroid hormone replacement therapy, numerous symptoms such as fatigue and muscle pain do not completely resolve in some patients with Hashimoto’s thyroiditis [18]. This phenomenon is hypothesized to be related to the ongoing autoimmune process in the thyroid gland. In a study, improvement in symptoms and a decrease in anti-TPO levels were observed following thyroidectomy in patients with HT whose symptoms had persisted despite becoming euthyroid with thyroid hormone replacement therapy [19]. Speckle echocardiography is more sensitive than conventional echocardiography in detecting LV dysfunction, and it has been suggested that speckle echocardiography should be a part of routine echocardiographic examinations due to its higher sensitivity to subtle myocardial changes in patients receiving cardiotoxic chemotherapy before an apparent change in LVEF occurs [20]. Moreover, contrary to other echocardiographic variables, a lower global subendocardial/subepicardial strain ratio and lower global endocardial strain are significantly associated with dyspnea [21]. Likewise, in our study, despite detection of normal ejection fractions with conventional echocardiography in HT patients after they have become euthyroid, their global strain values remained lower, similar to those of the hypothyroid HT group. In addition, low global strain was correlated with anti-TPO values in both hypothyroid and euthyroid HT patients. In studies examining LV strain in patients with subclinical hypothyroidism and elevated anti-TPO, lower global strain values were found when compared to healthy controls [22].

In our investigation, we observed a higher proportion of female patients, indicating a predominance of the female gender among individuals diagnosed with Hashimoto’s disease. The occurrence of systemic autoimmune disorders exhibits a gender bias, with a greater prevalence in women [23]. These conditions frequently result in the dysfunction of organs and tissues, with particular impacts on the myocardial microvasculature, which can lead to the onset of inflammatory cardiomyopathies and the progression of atherosclerotic diseases in female patients. Chronic inflammation is recognized as a significant contributor to heart failure in those with autoimmune conditions [24]. Additionally, longitudinal strain is often compromised in various diseases, including rheumatoid arthritis and systemic lupus erythematosis, making the monitoring of patients with declining global longitudinal strain (GLS) values crucial in this context [25,26].

TIMP-1 is one of the key regulatory molecules involved in extracellular matrix remodeling. TIMP-1 plays a role in hypertension, LV hypertrophy, atherosclerosis, and coronary plaque rupture [27,28].

A research investigation revealed that autoimmune-mediated dysthyroidism is linked to elevated levels of vascular cell adhesion molecule 1 (VCAM-1), intercellular adhesion molecule-1 (ICAM-1), and TIMP-1 in peripheral blood when compared to cases of non-autoimmune thyroid disease [29]. Notably, this study reported no alterations in the levels of inflammatory biomarkers, even following the treatment of patients with hypothyroidism, a finding that aligns with the results of our own research. TIMP-1 also has cytokine-like activity independent of matrix metalloproteinases. This characteristic allows TIMP-1 to actively participate in several essential biological processes, including cell proliferation, differentiation, apoptosis, and angiogenesis [30]. It was reported that TIMP-1 might be used as a biomarker in predicting cardiovascular events and all-cause mortality [31]. TIMP-1 also plays an active role in myocardial fibrosis and in the onset of pathological processes at an early stage, before the development of heart failure. In a research investigation involving individuals diagnosed with type 2 diabetes mellitus, a significant correlation was observed between late gadolinium enhancement on cardiac MRI and serum levels of TIMP-1. This finding underscores the potential of TIMP-1 as a predictive biomarker for cardiac fibrosis [32]. Similarly, in our study, higher TIMP-1 levels were found in both hypothyroid and euthyroid HT patients compared to the control group. TIMP-1 level was negatively correlated with LV global strain in both HT groups. This may be explained by the autoimmune component of Hashimoto’s disease. As a matter of fact, TIMP-1 levels were positively correlated with anti-TPO levels in both hypothyroid and euthyroid HT patients. In a recent study by our team, low global strain values were found in both hypothyroid and euthyroid patients with Graves’ disease and were correlated with TIMP-1 [33]. Therefore, the autoimmune component of the disease rather than the deficiency or excess of thyroid hormone may be the determining factor in patients with Hashimoto’s thyroiditis.

In conclusion, Hashimoto’s thyroiditis is associated with impaired segmental and global longitudinal LV strain, and this impairment is correlated with TIMP-1 and anti-TPO levels, indicating the involvement of cardiac tissue beyond the thyroid gland. These changes seem to be independent of thyroid hormone levels, indicating the autoimmune aspect of the disease.

## Figures and Tables

**Table 1:** Baseline characteristics of the study population.

	Total (*n* = 122)	Control ^1^ (*n* = 40)	Hypothyroid ^2^ HT (*n* = 40)	Euthyroid ^3^ HT (*n* = 42)	*p* Value	Post Hoc Comparisons’ *p* Values		
Age, years	28.3 ± 7.7	26 ± 2.5	28.4 ± 7.8	30.2 ± 9.2	0.154			
Female *n* (%)	107 (87.7)	34 (85)	35 (87.5)	38 (90.5)	0.774			
Heart rate, bpm		72 ± 10	68 ± 10	74 ± 8	0.872	N/A		
Systolic blood pressure, mmHg		125 ± 12.5	115 ± 10.5	124 ± 11.4	0.895	N/A		
Diastolic blood pressure, mmHg		73.5 ± 7.4	70.3 ± 6.8	72.8 ± 7.2	0.925	N/A		
Arrhythmia		3 (0.075)	4 (0.01)	3 (0.071)	0.826	N/A		
TFT						p ^1,2^	p ^1−3^	p ^2,3^
*T*_3_ (ng/L)		3.2 (0.9)	1.2 (0.4)	3.2 (0.9)	<0.001	<0.001	1.00	<0.001
*T*_4_ (ng/L)		1.0 (0.3)	0.3 (0.2)	1.0 (0.3)	<0.001	<0.001	1.00	<0.001
TSH (mU/L)		1.9 (0.9)	14.3 (14.3)	1.9 (2.0)	<0.001	<0.001	1.00	<0.001
TIMP 1 (ng/mL)		5.4 (2.0)	9.8 (3.9)	8.4 (4.6)	<0.001	<0.001	<0.001	1.00

HT, Hashimoto’s thyroiditis; TFT, thyroid function tests; N/A: Not Available, TIMP-1, tissue inhibitor of metalloproteinase-1; p ^1,2^ shows *p* value for comparison of control group vs. hypothyroid HT patients, p ^1−3^ shows *p* value for comparison of control group vs. euthyroid HT patients, p ^2,3^ shows *p* value for comparison of hypothyroid vs. euthyroid HT patients.

**Table 2:** Comparisons of echocardiographic indices among the groups.

Measurement	Controls (*n* = 40)	Hypothyroid (*n* = 40)	Euthyroid (*n* = 42)	*p* Value	p ^1,2^	p ^1–3^	p ^2,3^
Left atrial diameter (mm)	35 (3)	36 (3)	35 (2)	<0.001	<0.001	0.011	0.247
Left atrial volume index (ml/m^2^)	31 (1)	39 (2)	33 (1)	<0.001	<0.001	0.123	<0.001
Aortic root (mm)	31 (1.8)	32 (3)	32 (2)	0.368	-	-	-
LVEDD (mm)	44 (5)	48 (4)	47 (4)	<0.001	<0.001	0.007	0.029
LVESD (mm)	33 (2)	33 (1)	32 (2)	<0.001	0.005	0.986	<0.001
LVEF (%)	60 (6.5)	58 (4.8)	59 (4)	0.025	0.027	0.168	1.00
IVS. (mm)	0.9 (0.2)	1 (0.2)	1 (0.1)	0.012	0.063	0.018	1.00
PW thickness (mm)	0.7 (0.1)	0.9 (0.1)	0.8 (0.1)	<0.001	<0.001	0.068	<0.001
E-wave (cm/s)	70.5 (12)	66 (12)	70 (7)	0.005	0.033	1.00	0.007
A-wave (cm/s)	60 (5)	66 (5)	65 (10)	<0.001	<0.001	<0.001	0.236
E’	10.2 (1.1)	7.9 (1.4)	9.5 (1.3)	<0.001	<0.001	0.200	<0.001
IVRT (ms)	83 (9)	123 (15)	88 (38)	<0.001	<0.001	0.020	<0.001
DT (ms)	197 (13)	226 (13)	203 (22)	<0.001	<0.001	0.126	<0.001
E/A	1.2 (0.2)	1.0 (0.1)	1.1 (0.1)	<0.001	<0.001	0.077	<0.001
E/E’	7.1 (1.6)	8.2 (2.0)	7.5 (0.9)	<0.001	<0.001	0.111	0.069

LVEDD, left ventricular end-diastolic diameter; LVESD, left ventricular end-systolic diameter; IVS, interventricular septum; IVRT, isovolumic relaxation time; DT, deceleration time; LVEF, left ventricular ejection fraction. p ^1,2^ shows *p* value for comparison of control group vs. hypothyroid HT patients, p ^1–3^ shows *p* value for comparison of control group vs. euthyroid HT patients, p ^2,3^ shows *p* value for comparison of hypothyroid vs. euthyroid HT patients.

**Table 3:** Comparisons of the left ventricular longitudinal strain values among the groups.

Region	Basal/Mid	Controls (*n* = 40)	Hypothyroid HT (*n* = 40)	Euthyroid HT (*n* = 42)	*p* Value	p ^1,2^	p ^1–3^	p ^2,3^
						p	p	p
Posterior Septal Wall	Basal	−18 (6)	−16 (8)	−17 (6)	0.065	-	-	-
	Mid	−21 (6)	−17 (7)	−17 (6)	0.001	0.004	0.008	1.00
Anterior Septal Wall	Basal	−18 (5)	−14 (6)	−16 (5)	0.002	0.002	1.00	0.038
	Mid	−20.5 (5)	−17 (5)	−18 (4)	<0.001	<0.001	0.003	1.00
Lateral Wall	Basal	−18 (4)	−15 (6)	−17.5 (7)	0.002	0.003	1.00	0.031
	Mid	−20 (4)	−17 (5)	−19 (6)	0.002	0.002	0.161	0.361
Inferior Wall	Basal	−19 (3)	−14 (8)	−18 (6)	<0.001	<0.001	0.517	0.016
	Mid	−21 (3)	−17 (5)	−20 (4)	<0.001	<0.001	0.046	0.045
Anterior Wall	Basal	−18 (3.8)	−13.5 (5)	−14.5 (7.4)	<0.001	<0.001	<0.001	0.492
	Mid	−20.5 (2.8)	−14.5 (6.8)	−16 (7)	<0.001	<0.001	<0.001	1.00
Apical Regions	Anterior Apex	−26 (4)	−21 (8)	−21 (7)	<0.001	<0.001	<0.001	1.00
	Inferior Apex	−26 (4)	−22 (9)	−22 (7)	<0.001	<0.001	<0.001	1.00
	Lateral Apex	−26 (5)	−22 (8)	−22 (7)	<0.001	0.002	0.003	1.00
	Apical Cap	−27 (5)	−21 (7)	−21 (6)	<0.001	<0.001	<0.001	1.00
GLS Values	GLS A3C	−20.4 (2.1)	−18.6 (4.4)	−17.9 (6.1)	<0.001	0.003	0.003	1.00
	GLS A4C	−20.4 (2.3)	−17.8 (4.4)	−18.3 (3.8)	<0.001	<0.001	<0.001	1.00
	GLS A2C	−21.2 (2.7)	−16.2 (3.9)	−18.7 (3)	<0.001	<0.001	<0.001	0.081
	LVGLS	−20.5 (4.4)	−17.5 (3.0)	−18.4 (3.1)	<0.001	<0.001	<0.001	0.958

GLS, global longitudinal strain; A3C, apical 3 chamber; A4C, apical 4 chamber; A2C, apical 2 chamber, LVGLS, left ventricular global longitudinal strain; p ^1,2^ shows *p* value for comparison of control group vs. hypothyroid HT patients, p ^1–3^ shows *p* value for comparison of control group vs. euthyroid HT patients, p ^2,3^ shows *p* value for comparison of hypothyroid vs. euthyroid HT patients.

## Data Availability

The data presented in this study are available on request from the corresponding author due to privacy/ethical restrictions.
